# Toward Improving Medication Adherence: The Suppression of Bitter Taste in Edible Taste Films

**DOI:** 10.1155/2018/8043837

**Published:** 2018-06-25

**Authors:** Silvy Cherian, Brian Sang Lee, Robin M. Tucker, Kevin Lee, Gregory Smutzer

**Affiliations:** ^1^Department of Biology, Temple University, 1900 N. 12th Street, Philadelphia, PA 19122, USA; ^2^Department of Food Science and Human Nutrition, Michigan State University, East Lansing, MI 48824, USA

## Abstract

Bitter taste is aversive to humans, and many oral medications exhibit a bitter taste. Bitter taste can be suppressed by the use of inhibitors or by masking agents such as sucralose. Another approach is to encapsulate bitter tasting compounds in order to delay their release. This delayed release can permit the prior release of bitter masking agents. Suppression of bitter taste was accomplished by encapsulating a bitter taste stimulus in erodible stearic acid microspheres, and embedding these 5 *µ*meter diameter microspheres in pullulan films that contain sucralose and peppermint oil as masking agents, along with an encapsulated masking agent (sucralose). Psychophysical tests demonstrated that films which encapsulated both quinine and sucralose produced a significant and continuous sweet percept when compared to films without sucralose microspheres. Films with both quinine and sucralose microspheres also produced positive hedonic scores that did not differ from control films that contained only sucralose microspheres or only empty (blank) microspheres. The encapsulation of bitter taste stimuli in lipid microspheres, and embedding these microspheres in rapidly dissolving edible taste films that contain masking agents in both the film base and in microspheres, is a promising approach for diminishing the bitter taste of drugs and related compounds.

## 1. Introduction

Taste plays a critical but often underappreciated role in dispensing medications to young children [1–3]. Antibiotics are among the most commonly prescribed drugs to children, and antibiotics such as amoxicillin exhibit a bitter taste in the oral cavity [4, 5]. Because many young children are unable or unwilling to swallow capsules, caplets, or tablets due to their fear of choking [1, 6], some workers suggest crushing the tablet [7]. However, this action may cause an increase in bitter taste intensity since the nonbitter coating of the encapsulated drug is compromised. The poor palatability and bitter taste of many orally administered medications often result in avoidance of those drugs [1, 3].

Young children are often more sensitive to bitter taste than older children and adults [8, 9]. Recent psychophysical data suggest that negative responses to many bitter tasting compounds increase postnatally or early in childhood development [10–13]. These responses are likely due to anatomical differences as children possess higher densities of fungiform papillae and taste pores [14]. The dislike of bitter tasting medications, and lack of pleasant tasting drug formulations for young children, may lead to suboptimal treatment in these individuals [3].

One approach to minimize the bitter taste of a drug is to chemically block a specific bitter taste receptor [15, 16]. In humans, bitter taste is activated by a family of twenty-five G-protein-coupled taste receptors that are encoded by the *TAS2R* gene family [17]. A number of bitter taste antagonists have recently been identified and include compounds such as probenecid, GIV3727, and γ-aminobutyric acid [18]. However, bitter taste blockers often show narrow specificity [16], may not fully block a bitter taste receptor, or may be specific to only one of several bitter taste receptors that might be activated by a specific compound or drug. This approach may result in only a partial blockage of bitter taste.

Another approach to improve drug palatability is to mask bitter taste with an excipient or flavor enhancer. Masking the bitter taste of prescription drugs can be accomplished by adding flavors, sweeteners, or effervescent agents [19–22]. A variety of bitter taste masking agents have been reported and include compounds such as sodium gluconate and monosodium glutamate [18]. Split-tongue taste stimulation studies have further demonstrated that bitter taste is suppressed by sweet taste stimuli [23], and sweet taste stimuli have the added benefit of reducing pain in infants, children, and adults [22, 24]. In a study involving taste mixtures, the sweet taste of sucrose was the strongest suppressor (and the least suppressed) of other taste qualities, including bitter taste [25]. Other masking techniques include coating the drug with an insoluble polymer matrix, complexing the drug with cyclodextrins, or using prodrugs that have decreased bitter taste [26, 27].

A drug delivery system that provides a therapeutic agent in the correct dosage, in a manner that optimizes efficacy, minimizes bitter taste perception, and decreases choking hazards, is predicted to increase drug compliance in young children [28, 29]. One promising approach to this problem is to encapsulate a bitter tasting compound within fatty acid microspheres [30, 31].

Stearic acid is a promising fatty acid for encapsulating drugs because this saturated long-chain biocompatible fatty acid exhibits minimal taste [32], functions as a solid carrier for drugs [33], melts at a temperature that is suitable for encapsulating compounds [31], and is resistant to decomposition at high temperatures [34]. In addition, dietary stearic acid has a neutral effect on serum low-density lipoprotein levels [35]. Since free fatty acids are not appreciably hydrolyzed in the oral cavity, the encapsulated compound (drug) must undergo surface and/or bulk erosion before it is released [32]. During erosion, the bitter taste of the released compound can be efficiently masked by pleasant tasting stimuli that are more rapidly released from edible taste strips as these strips dissolve upon contact with the oral mucosa. In addition, the encapsulation of drugs for incorporation into rapidly dissolving films will minimize choking hazards in both young children and the elderly. Finally, the delayed erosion of microspheres that contain sweet taste stimuli can further mask bitter taste.

Quinine hydrochloride (quinine) is a naturally occurring compound that is isolated from the bark of *Cinchona* trees, or from *Remijia* plants [36]. This compound elicits a strong bitter taste in humans [37], has a melting point that is suitable for encapsulation in lipid microspheres, is easily assayed in the lab, and is a useful model for bitter tasting drugs [38]. This alkaloid has been used to treat malaria and babesiosis for over one hundred years [39, 40]. In this study, quinine was used as a representative bitter-tasting drug in order to identify mechanisms that mask its bitter taste. Quinine was then encapsulated within lipid microspheres so that the masking effects of unencapsulated and encapsulated sweeteners and peppermint oil could be identified.

In this report, a rapidly dissolving edible taste strip is described that is formulated with sucralose and is flavored with peppermint. This edible strip composition is then used for embedding lipid microspheres that contain quinine, sucralose, or no taste stimulus. We then demonstrate that encapsulating and masking the bitter taste of quinine in sucralose-peppermint taste strips that also contain encapsulated sucralose, is a promising two-step approach to minimize bitter taste perception in the human oral cavity.

## 2. Methods

### 2.1. Chemicals and Reagents

Food grade pullulan (*α*-1,4-; *α*-1,6-glucan) was obtained from NutriScience Innovations, LLC, Trumbull, CT; food grade hydroxypropyl-methylcellulose (HPMC) was obtained from Dow Chemical Co., Midland, MI; and white food coloring was obtained from LorAnn Oils (Lansing, MI). Glycerol was obtained from Fisher Scientific. Sucralose was obtained from Tate & Lyle (MacIntosh, AL), HEPES, quinine HCl·2H_2_O, and tert-butylhydroquinone (TBHQ) were obtained from Sigma-Aldrich (St. Louis, MO), D-mannitol was obtained from CalBiochem, and peppermint oil was obtained from Terralyn (Philadelphia, PA). Xanthan gum was obtained from Penn Herbs Inc. (Philadelphia, PA), water was obtained from Deer Park (Stamford, CT), and food grade stearic acid was obtained from Loudwolf Industrial and Scientific (Dublin, CA). Glycerol, HEPES buffer, and phosphate buffer were sterilized before use.

### 2.2. Preparation of Stearic Acid Microspheres That Encapsulated No Stimulus, Encapsulated Quinine, or Encapsulated Sucralose

A modification of the hot melt method was used to prepare stearic acid microspheres [31] that encapsulated no taste stimulus, encapsulated quinine HCl·2H_2_O (m.p. = 115°C), or encapsulated sucralose. For quinine microspheres, quinine and stearic acid were combined at a 5.5 : 1 wt./wt. ratio of lipid to taste stimulus. The mixture was ground to a fine powder, fully melted at 116–118°C in a mineral oil bath before the mixture was added to HEPES buffer. The sample was mixed thoroughly with a spatula, poured into a rapidly stirring solution of 5 mM HEPES buffer at pH 8.0 at 60–65°C, and cooled for 15 minutes with rapid stirring (∼3000 RPM). The resulting microspheres were collected by centrifugation at 7500 x g for 10 minutes at 15°C. The soft pellet was resuspended in a small volume of HEPES buffer and washed with HEPES buffer by vacuum filtration. Microspheres were again washed with HEPES buffer at pH 8.0 and finally with a rinse of sterile water or 2.5 mM phosphate buffer at pH 7.0. Microspheres were dried overnight in a vacuum oven at 30°C or air dried in the dark at room temperature for 24 hours. Dried microspheres were stored at −11°C in tightly sealed containers until use. Microspheres that encapsulated no taste stimulus (empty microspheres) were prepared by the same procedure except at a melting temperature of 100°C.

The encapsulation of sucralose occurred in a similar fashion except 5 mM sodium acetate buffer at pH 4.15 or 4.25 was used to prepare microspheres. A 5.5 : 1 weight ratio of stearic acid and sucralose powder was prepared, ground in a cold mortar and pestle, added to a 30 ml beaker, and heated to 116–118°C to fully melt sucralose at a temperature just below the decomposition temperature of sucralose [41]. The off white solution was poured into a rapidly stirring solution of sodium acetate buffer at 60–65°C. Microspheres were centrifuged, washed, and dried similar to that of quinine microspheres.

### 2.3. Preparation of Edible Films That Contain Bitter Taste Masking Agents

Rapidly dissolving edible taste films were prepared as previously described [32, 37, 42]. Briefly, pullulan was combined with the polymer hydroxypropyl-methylcellulose at a wt./wt. ratio of 11.5 : 1 at a final aqueous polymer concentration of 3.00%. In addition, xanthan gum (0.05% wt./vol) was added as a thickening agent [43], and D-mannitol (3.3 mM) was used as a humectant and sweetener [44, 45]. The polymer solution also included sucralose, and ethanol-free peppermint oil as masking agents, along with the antioxidant TBHQ (0.005% w/v).

Sucralose was added to edible taste films at an amount that was previously determined by a sweet taste preference test [46]. Peppermint oil was added at its maximal solubility in the polymer solution (0.004% v/v). In addition, 0.05% glycerol was added as a plasticizer. Finally, white food coloring (0.012%) was added to aid in visualizing edible films and to partially mask the appearance of microspheres in the dried films.

Three of the four edible film formulations contained lipid microspheres. For these films, two hundred milligrams of dried microspheres were mixed with 40 ml of the polymer solution described above in nonstick tubes. The mixture was vortexed, warmed to 30°C, and sonicated with an Ultrasonic Processor Model GE50T horn sonicator for four times for ten seconds each at 50% intensity. Then, 8.80 ml of the mixture was placed in weigh boats previously washed with 70% ethanol, and allowed to dry for 24–36 hours at room temperature in the dark on a level surface. After drying, the films were cut into one-inch films and stored at −11°C in sealed containers. Films were used within four weeks of preparation.

The three taste film formulations that contained microspheres (formulations 2–4) underwent statistical analysis to compare effectiveness. These three formulations differed in microsphere content but contained identical amounts of sucralose and peppermint in the pullulan film base. See Table 1 for a description of the four film formulations that were used in this study. In addition to the edible film that contained no microspheres, these formulations included films with empty (blank) microspheres only, films with quinine microspheres and empty microspheres, and films with both quinine and sucralose microspheres. Formulations 2–4 were composed of edible strips that contained the same amount of lipid microspheres per strip.

### 2.4. Microscopic Analysis of Microspheres and Edible Films

Scanning electron microscopy (SEM) images of microspheres were obtained with a Quanta 450FEG (FEI Co.) SEM with secondary and backscatter detectors at the College of Engineering Nano Instrumentation Center at Temple University.

### 2.5. Spectroscopic Assays for Quinine HCl

The quinine content of microspheres was identified by dissolving microspheres in 80% acetonitrile/20% HEPES at pH 8.0 for absorption measurements at 329 nm. For fluorescence measurements of quinine, emission was obtained in a PTI fluorometer (Horiba Scientific) in 90% acetone and 10% 0.5 M H_2_SO_4_. Excitation wavelength was 320 nm, and emission wavelength was 440 nm.

### 2.6. Infra-Red Spectroscopy and Melting Temperature Determination

Fourier transform infra-red spectra (FT-IR) of microspheres was undertaken to identify potential degradation of stearic acid, quinine, or sucralose and to determine whether microspheres encapsulated quinine or sucralose. FT-IR spectra of microspheres with no encapsulated compound, sucralose-containing microspheres, and quinine-containing microspheres were obtained with a Thermo Scientific Nicolet iS5 spectrometer (Waltham, MA). The spectra were obtained in a range between 4000 cm^−1^ and 400 cm^−1^. Finally, the melting temperature of dried microspheres was obtained with a Mel-Temp II capillary melting point apparatus (Holliston, MA).

### 2.7. Subject Population

For this pilot study, a total of 15 healthy volunteers participated in the psychophysical evaluations of all four strip formulations. The same 15 subjects participated in all four psychophysical studies. The average age of test subjects was 22.3 ± 1.3 years (range: 18–65), and 40% of study participants were males. In terms of race and ethnicity, 70.0% of subjects were Asian, 23.3% were Caucasian, 3.3% were Black/African American, and 3.3% were of Hispanic descent.

### 2.8. Psychophysical Taste Studies with Edible Strip Formulations

Subjects were asked to refrain from eating or drinking for 30 minutes prior to testing sessions. Subjects with diabetes, neurological disorders, or who had recent dental visits, were excluded from this study. All subjects were healthy by self-report. Study subjects were recruited through flyers and by word of mouth. The study protocol was approved by the Institutional Review Board at Temple University, and all study participants provided written informed consent. Finally, the subjects were reimbursed for their time.

The general Labeled Magnitude Scale (gLMS) was used for all suprathreshold intensity measurements [47]. This higher order polynomial scale contains labels for barely detectable (1.4), weak (6.0), moderate (17.0), strong (34.7), very strong (52.5), and strongest imaginable sensation of any kind (100.0). All test subjects were trained in the use of the gLMS [39] by asking them to rate intensities of imagined or remembered sensations that included both gustatory and nongustatory stimuli [47].

For taste quality measurements, subjects were presented with a simplified list and asked to choose from the following taste qualities: sweet, bitter, other taste, or no discernable taste. For taste quality measurements, up to two choices were allowed for each response. For Temporal Dominance of Sensation analysis (see below), the first predominant taste quality was used. An overall hedonic response for each film was then obtained after intensity and taste quality measurements were identified for each taste film. For hedonics ratings, the degrees of liking-disliking of microsphere-containing edible films were rated on a horizontal (bipolar) hedonic gLMS (0 = neutral; ±6.0 = weakly like/dislike; ±17.0 = moderately like/dislike; ±34.7 = strongly like/dislike; ±52.5 = very strongly like/dislike; and ±100.0 = strongest imaginable like/dislike of any kind) according to Duffy et al. [48].

Both oral instructions and a photograph were used to describe the uniform placement of edible films on the tongue. For uniform placement of films on the tongue, all subjects practiced with a control taste film before the start of the experiment. This taste film contained only pullulan and hydroxypropyl methylcellulose. During this time, subjects were instructed to place a film on the front center of their tongue and touch the tongue to the roof of their mouth to instantly dissolve the thin film [37]. Each subject was instructed to signal the test administrator as soon as the taste film came in contact with the roof of the mouth (time zero) by raising his or her hand. Subjects were instructed to report a numerical taste intensity value and a taste quality response at 10-second intervals from 0 to 120 seconds. The subjects were then asked to report a hedonic value for the edible taste strip. Each trial of two strips consisted of a control film with no taste stimulus in the film base and one of the four experimental films described above (Table 1). The presentation of the two films for each trial was randomized, and each trial was repeated (four strips per formulation). The presentation of the four different strip formulations was also randomized.

### 2.9. Sucralose Assay

Sucralose content of microspheres was assayed by the procedure of Youssef et al. [49] except that a stock concentration of 5.5 mM KMnO_4_ was used, and the incubation time was extended to 35 minutes. For the assay, sucralose and sucralose-containing microspheres were fully dissolved in HPLC-grade acetonitrile. The final reaction volume was 10.775 mls. The reaction was allowed to proceed at room temperature in the dark with gentle shaking. Samples were vortexed and then centrifuged for 3-4 minutes at the maximum speed in a clinical centrifuge to separate undissolved stearic acid from the assay solution. The clumped stearic acid was carefully removed from the top of the assay solution with a spatula. Absorbance of the clear solution was measured at 610 nm in a Pharmacia Ultrotech 2000 spectrophotometer.

### 2.10. Statistical Analysis

Psychophysical data were analyzed using IBM SPSS Statistics version 24 and Microsoft Excel. Significance was defined as *p* < 0.05. The two trials for each treatment were averaged. All data are presented as means ± standard error of the mean (SEM). Repeated measures analysis of variance (RMANOVA) and pairwise comparisons with Bonferroni corrections were used to evaluate differences between the treatments. Intensity and hedonics were measured at 10 second intervals as described above. Persistence was measured as the elapsed time until the gLMS intensity rating was <1. Temporal Dominance of Sensations (TDS) methodology was used to characterize how the perception of the dominant taste quality changed over time [50, 51]. For TDS analysis, in order to determine whether the different percepts were significant as opposed to randomly occurring, a total of 12 possible attributes were possible (100% sweet, bitter, other, or no taste; 75% sweet, bitter, other, or no taste; or 50% sweet, bitter, other, or no discernible taste). According to the method discussed by Pineau and Schilch [51], using the averaged values of the two replications, significance was determined by the results of a binomial test with *p*=1/12, 15 trials, and *α*=0.05 and divided by the number of trials, giving a significance level to ratings of 26.7% and above. Using G^∗^Power 3.1, given the repeated measures design, 15 participants were needed to achieve 90% power, assuming a large effect size and setting alpha = 0.05 [52].

## 3. Results

### 3.1. Preparation of Quinine and Sucralose Microspheres

The bitter taste stimulus quinine HCl was successfully encapsulated within stearic acid microspheres by the hot melt method in pH 8.0 buffer [31]. This hydrophobic compound produced a median wt./wt. ratio of stearic acid to quinine content of 11.1 : 1 ± 1.2 in microspheres (*n*=4).

With pH 8.0 buffer, no sucralose was incorporated into stearic acid microspheres. A buffer pH of 5.20 yielded low amounts of sucralose encapsulation (86.6 : 1 wt. ratio of stearic acid to sucralose, *n*=2). An acidic buffer pH of 4.15 or 4.25 improved encapsulation efficiency so that sucralose microspheres yielded a median wt/wt ratio of 35.4 : 1 (*n*=5).

**Figure 1:**
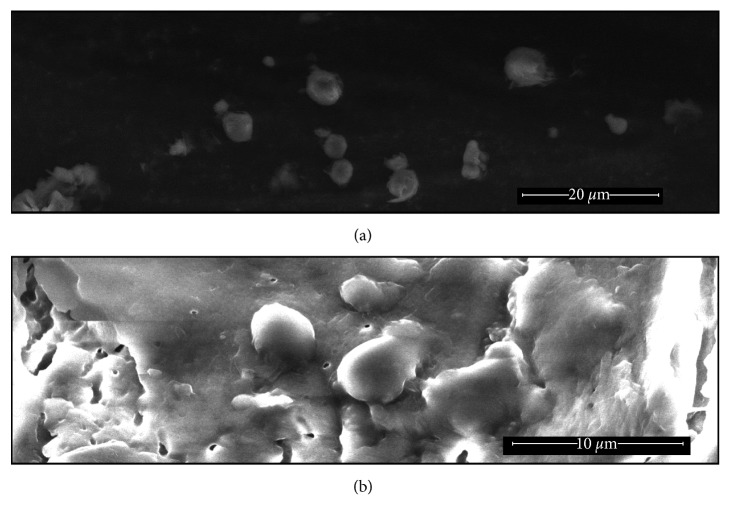
Scanning electron microscopy (SEM) images of stearic acid microspheres that encapsulated quinine HCl. Lipid microspheres were prepared in pH 8.0 buffer at 65°C by the hot melt method. (a) Individual stearic acid microspheres that encapsulated quinine HCl. (b) Clusters of stearic acid microspheres that encapsulated quinine HCl.

Scanning electron microscopy (SEM) was used to observe the surface, shape, and size of stearic acid microspheres.  Figure 1 shows a scanning electron micrograph image of stearic acid microspheres that contained quinine HCl. Microspheres that encapsulated quinine HCl appeared spherical in shape, with a median diameter of 3.1 ± 0.2 *µ*meters (*n*=21). Quinine HCl microspheres also formed large clusters that were apparent in SEM images.

**Figure 2:**
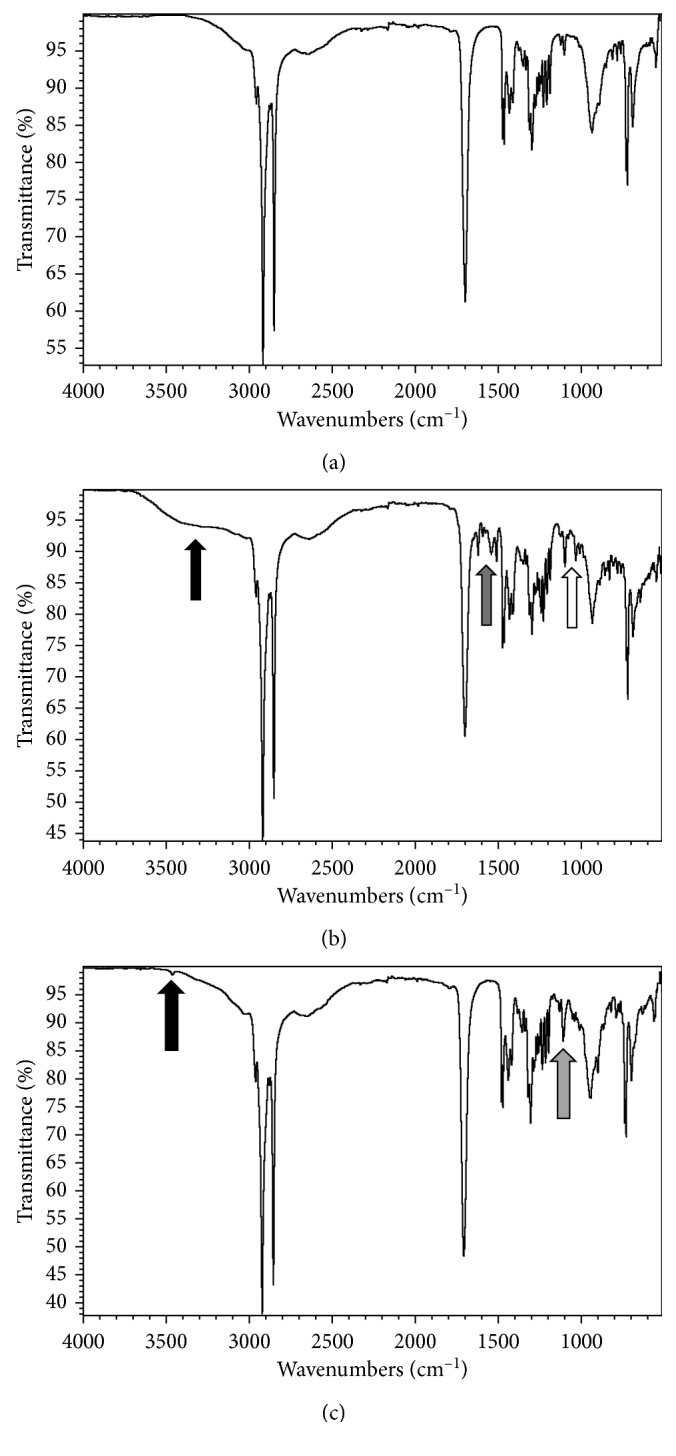
FT-IR spectra of lipid microspheres. The *y*-axis represents % transmittance, and the *x*-axis represents wavenumber in cm^−1^. The *y*-axis is scaled to the longest downward transmission peak in all four panels. (a) Stearic acid microspheres with no encapsulated compound. (b) Stearic acid microspheres that encapsulated quinine by the hot melt method. Black vertical arrow represents IR band at 3400 cm^−1^, gray arrow represents IR band at 1600 cm^−1^, and open arrow represents an IR band at 1050 cm^−1^. (c) Stearic acid microspheres that encapsulated sucralose by the hot melt method. Black arrow represents band at 3500 cm^−1^, and gray arrow represents band near 1100 cm^−1^. See Results section for identification of IR bands.

Figure 2 shows representative FT-IR spectra of stearic acid microspheres that encapsulated no taste stimulus and stearic acid microspheres that encapsulated quinine or sucralose. For empty microspheres, strong infrared bands at 2933 and 2864 cm^−1^ are assigned to C-H stretching, and the strong peak at 1700 cm^−1^ corresponds to the carbonyl group stretch of stearic acid. The smaller peaks at 1250 cm^−1^ correspond to stretching peaks of the carboxylic acid group of stearic acid [34, 53]. Finally, a broad hydroxyl group bending is seen at 943 cm^−1^ [54].

Figure 2(b) is an infra-red spectrum of quinine-containing microspheres. Figure 2(b) indicates the presence of low amounts of quinine as indicated by the broadening of the peak near 3200−3300 cm^−1^ (-OH stretch of quinine backbone, black arrow) and the appearance of a band at 1650 cm^−1^ (C=C stretch, (aromatic, alkene), gray arrow), and at 1100 cm^−1^ (C-O stretch (ether), open arrow) [55, 56].  Figure 2(c) is an infra-red spectrum of sucralose-containing microspheres. A small band is observed at 3500 cm^−1^ (-OH stretch of sucralose, dark arrow), and a band at 1000 cm^−1^ in the fingerprint region matches published IR spectra of sucralose (open arrow) [41].

Figures 2(b) and 2(c) both show a sharp peak at 1700 cm^−1^ and indicate the presence of carbonyl groups in stearic acid microspheres. Figures 2(b) and 2(c) both display a broad -OH bend [53] at 943 cm^−1^ and suggest that a population of stearic acid molecules is protonated in sucralose and quinine microspheres.

The IR spectra in Figure 2 also indicate that stearic acid did not undergo decomposition during microsphere preparation and that most of the IR signal in microspheres arose from the stretching and bending of stearic acid functional groups. The IR data from quinine and sucralose microspheres further supports chemical and spectral assays of these loaded microspheres, which indicated that microspheres were primarily composed of stearic acid (>90% by weight).

Finally, stearic acid microspheres that contain no encapsulated compound (empty microspheres) exhibited a melting point range of 55 to 60°C while quinine-containing microspheres prepared at a wt. : wt. ratio of 5.5 : 1 stearic acid melted at a temperature range of 55 to 58°C. Sucralose-containing microspheres melted over a range of 48–53°C. These melting point ranges are similar to those reported with stearic acid microspheres that encapsulated the cephalosporin antibiotic cefuroxime axetil [30]. These melting temperature ranges indicate that microspheres prepared by the hot melt method in HEPES or sodium acetate buffer exhibited melting point ranges that were lower than the compounds from which they were prepared. Finally, encapsulated quinine or encapsulated sucralose did not significantly depress the melting range of microspheres when compared to empty microspheres that only contained steric acid.

### 3.2. Psychophysical Taste Studies with Edible Films That Contained No Microspheres (Formulation One)

Table 1 summarizes the taste stimulus components of all four strip formulations. Before experiments with microsphere-containing films were completed, taste studies were finalized with edible films that contained both sucralose and peppermint oil as integral components of the film base, but contained no lipid microspheres. Formulation one represented baseline taste intensity and taste quality responses in the absence of any encapsulated compounds, and identified the time window that these masking agents could be perceived immediately after the taste strips dissolved. Also, this formulation yielded hedonic data for edible films that contained no microspheres.

### 3.3. Edible Film Formulations That Contained Microspheres (Formulations 2, 3, and 4)

A weight ratio of 2 : 1 was chosen for sucralose and quinine microspheres because humans are more sensitive to bitter taste than to sweet taste [57, 58]. Also, lipid microspheres encapsulated hydrophobic quinine more efficiently than sucralose. In addition, all the formulations contained unencapsulated sucralose and peppermint oil in the film base (Table 2).

Edible strip formulations 2, 3, and 4 contained microspheres that were embedded within edible films (Table 1). For these three edible strip formulations, microspheres are predicted to undergo surface erosion and release their contents. This erosion is predicted to slightly delay the release of encapsulated compounds in the oral cavity. If so, this delay would allow the prior release of sucralose and peppermint oil from the edible film base into the oral cavity.

Formulation 2 was a control that contained only empty microspheres. The remaining two formulations (formulations 3 and 4) were tested in order to identify the added effect of encapsulated sucralose on further masking of the bitter taste of encapsulated quinine as these compounds are released into the oral cavity. The edible strip formulation that contained both quinine and sucralose microspheres (formulation 4) was predicted to release these two taste stimuli after the taste strips dissolved and as microspheres eroded in the oral cavity. If so, then edible strips that contained both quinine and sucralose microspheres should cause an decreased bitter taste response when compared to films that contained only encapsulated quinine along with empty microspheres (formulation 3). Statistical analysis of the psychophysical test results is described below.

### 3.4. Comparison of the Edible Film Formulations That Contained Microspheres

For statistical comparison of the three formulations that contained microspheres, the formulation that contained only empty microspheres was identified as the control. Measures of intensity, persistence, and hedonics (pleasantness) are presented in Table 3. While mean intensity ratings were higher for both quinine-containing microsphere films compared to control films with empty microspheres and no quinine, the maximum intensity score, persistence, and hedonic score of the quinine + sucralose microsphere film did not differ from either the control film with empty microspheres only or the quinine + empty microsphere film. The quinine + empty microsphere film persisted longer (*p*=0.005) and was rated less favorably (*p*=0.022) than the control film with only empty microspheres. Maximum taste intensity for all three films was rated between moderate to strong. Hedonic ratings for the quinine-empty microsphere film fell into the neutral to weakly dislike category while the other films were rated as weakly to moderately liked.

**Figure 3:**
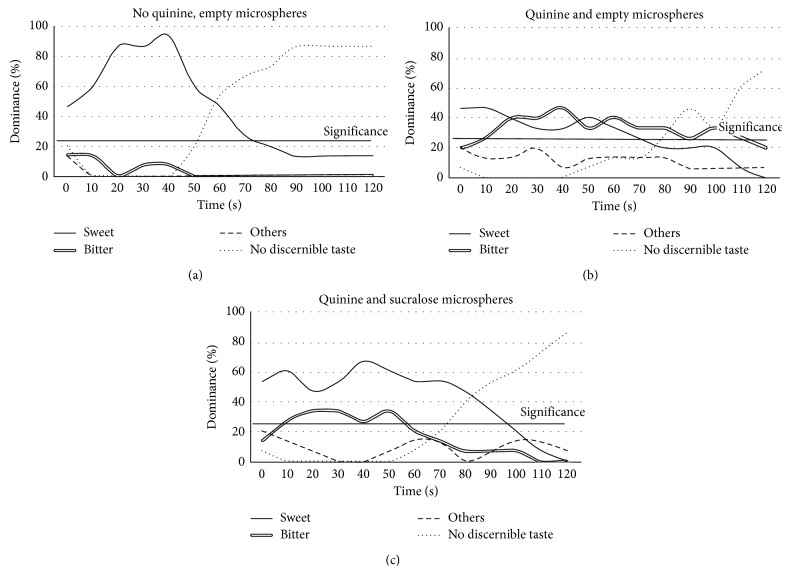
Temporal Dominance of Sensation (TDS) analysis indicated that the quinine microsphere plus sucralose microsphere film (c) was perceived as predominantly sweet (solid black line), and these perceptions more closely approximated those of the control film (a) compared to the quinine plus empty microspheres film (b). The bitterness (double black line) experienced in the films with sucralose plus empty microspheres (c) was reduced and persisted for less time when compared to the quinine plus empty microspheres film (b).

Temporal Dominance of Sensation analysis demonstrated differences in predominant taste sensation between the treatments (Figure 3). The control microsphere film with no quinine (empty microspheres) possessed a significant sweet taste quality for 70 seconds and then transitioned to no discernible taste (Figure 3). The two films with quinine (formulations 3 and 4) both elicited significant bitter taste perception. However, the quinine + sucralose microsphere films (Figure 3) had a higher perception of sweetness, and the bitter perception faded more quickly than the quinine + empty microsphere formulation (Figure 3) (∼55 s versus 100 s).

## 4. Discussion

This study is the first to successfully encapsulate the crystalline taste stimuli sucralose and quinine HCl within lipid microspheres. Sucralose is a polar compound that is difficult to encapsulate in lipid microspheres, and only one report of sucralose encapsulation by complex coacervation has been described [59]. In this report, we present simple protocols for encapsulating the noncaloric sweetener sucralose and quinine HCl in a naturally occurring lipid matrix.

Parallel encapsulation studies have shown that hydrophobic compounds such as the photosystem II herbicide 3-(3,4-dichlorophenyl)-1,1-dimethylurea (DCMU) (solubility in water is 0.042 g/l) are encapsulated at amounts that approach 13% of the total weight of microspheres (data not shown). Based on these results, hydrophobicity may be an important consideration for predicting encapsulation efficiency within stearic acid microspheres [60].

In addition to hydrophobicity, other parameters may affect encapsulation efficiency. These variables include the choice of buffer [31], aqueous buffer temperature, buffer pH, and the weight ratio of the encapsulated compound to stearic acid. Changes in encapsulation efficiency and drug release kinetics from microspheres could also be achieved by modifying the lipid composition of microspheres [61]. Possible lipid modifications include the addition of unsaturated fatty acids or oils and/or the inclusion of fatty acids of differing fatty acid tail length during microsphere formation. Also, the addition of small amounts of surfactants to the aqueous buffer may affect encapsulation efficiency [62]. These variables could also affect drug release kinetics from lipid microspheres. Finally, enteric coating of lipid microspheres may further modify the release characteristics of a drug from lipid microspheres [63].

In addition to altering encapsulation efficiency, the amount of encapsulated compound in edible films can be modified. This amount can be increased or decreased by varying the amount of microspheres that is added to the film solution, by preparing edible films that vary in size, or by preparing edible films that vary in thickness.

Our taste studies indicate that the inclusion of sweet taste stimuli and peppermint oil in thin, rapidly dissolving pullulan films, along with encapsulating sucralose in erodible microspheres, efficiently masked the bitter taste of quinine in the human oral cavity. Previous work has demonstrated that quinine-containing strips (with no microspheres) produced a moderate-to-strong bitter taste intensity [42]. In the current study, sucralose-peppermint films that also contained encapsulated quinine and encapsulated sucralose adequately blocked bitter taste. These films (formulation 4) were hedonically acceptable based on the totality of the psychophysical and TDS data. The quinine + sucralose microsphere film matched the control (empty microspheres and no quinine) film in terms of hedonic score, persistence, and maximum intensity. The mean hedonic score of quinine-sucralose microsphere films fell into the weakly to moderately like category; whereas, the quinine-empty microsphere films were rated as neutral to weakly dislike. While average intensity was higher for the quinine-containing films compared to control films, each film was rated as weakly to moderately intense, suggesting all were tolerable to participants.

The predominant taste quality for the quinine + sucralose microsphere film was sweet, while the predominant taste of the quinine + empty microsphere taste film varied between significantly bitter and sweet. Thus, sucralose microspheres successfully suppressed the bitterness associated with quinine to a significant degree. These studies with sucralose microspheres further support the hypothesis that bitter taste suppression is a general property of sweet taste stimuli. Bitter taste perception can be minimized and rapidly dissipated in the oral cavity by both masking and encapsulating bitter taste stimuli. This study is the first to utilize this novel two-step approach for masking and delivering bitter taste stimuli to the oral cavity.

In edible strips, peppermint oil exhibits a sweet balsamic taste that is masked by its distinct cooling effect in the oral cavity [64]. This cooling effect is likely caused by menthol [65, 66], which triggers the activation of cold-sensitive TRPM8 receptor/channels that localize to trigeminal neurons [67]. In addition, the analgesic properties of menthol are mediated through the selective activation of kappa opioid receptors [68]. Peppermint also produces a volatile odor that masks bitter taste perception in humans [69, 70]. Its inclusion in edible films likely contributed to the flavor enhancement of all four edible film preparations.

The mechanism of drug release from stearic acid microspheres into the oral cavity is complex, but may include diffusion of the drug through the outer hydrated layer of the lipid matrix, and followed by the erosion of microspheres by saliva [71]. This erosion of encapsulated taste stimuli may slightly delay their discharge into the oral cavity [28] when compared to the rapid release of masking compounds from rapidly dissolving, thin edible films. If so, then the rapid release of masking stimuli from pullulan-based films may efficiently minimize bitter taste perception before the encapsulated bitter taste stimulus is released from microspheres. In addition, the encapsulation of a compound (drug) in lipid microspheres will physically segregate this compound during storage and may protect that compound from aqueous environments.

## 5. Conclusions

Lipid microspheres show promise for advancing edible film technology as a drug delivery mechanism to the oral cavity. The encapsulation of compounds in lipid microspheres, and delivering these microspheres to the oral cavity by rapidly dissolving edible films that contain masking agents, may be promising approaches for masking bitter taste. This novel procedure should be useful for dispensing bitter tasting drugs to children, should benefit individuals with swallowing disorders, and should reduce choking hazards in the young and elderly. In addition, the amount of encapsulated compound can be readily modified in edible films. Future studies will optimize the encapsulation efficiency and release characteristics of drugs from fatty acid microspheres, and will explore the possibility of encapsulating additional masking compounds for minimizing bitter taste perception. These future studies will lead to the development of improved delivery methods for bitter tasting drugs and compounds.

## Figures and Tables

**Table 1:** Characteristics of the edible film and microsphere content of taste strip formulations.

Formulation	Edible film components	Microsphere content
1	Peppermint-sucralose film base	No microspheres
2	Peppermint-sucralose film base	Empty (blank) microspheres
3	Peppermint-sucralose film base	Quinine + empty microspheres
4	Peppermint-sucralose film base	Quinine + sucralose microspheres

For formulations 2–4, each one-inch square edible taste film contained 3.75 mg of lipid microspheres. Empty microspheres were included in formulations 2 and 3 so that microsphere density was uniformly maintained in formulations 2 through 4.

**Table 2:** Amount of sucralose and quinine HCl in the four edible strip formulations.

Formulation	Unencapsulated sucralose (all films) (nmoles)	Encapsulated sucralose (nmoles)	Total sucralose in edible strips (nmoles)	Encapsulated quinine (nmoles)
1	1060	0	1060	0
2	1060	0	1060	0
3	1060	0	1060	352
4	1060	178	1238	352

Formulation 1 contained no microspheres. The same batch of quinine microspheres was used in formulations 3 and 4.

**Table 3:** Measures of intensity, persistence, and hedonics of edible film formulations.

	Empty microspheres only, no quinine (control)	Quinine + empty microspheres	Quinine + sucralose microspheres	*p* value
Average intensity (gLMS)	6.5 ± 0.9^a^	11.4 ± 2.0^b^	10.2 ± 1.4^b^	<0.040
Maximum intensity	22.0 ± 3.0	25.9 ± 3.3	25.8 ± 3.1	N.S.
Persistence (s)	78.0 ± 5.9^a^	104.7 ± 5.6^b^	93.3 ± 5.6^a,b^	0.005
Hedonic score (units)	9.8 ± 1.7^a^	−1.6 ± 3.9^b^	6.3 ± 2.7^a,b^	0.022

Data are presented as means ± SE. Across each row, values with different superscripts are significantly different from each other. Average intensity for both the quinine and empty microspheres and the quinine and sucralose microspheres were higher than the control film that contained empty microspheres only, but did not differ from each other. The taste of the quinine and empty microsphere film persisted longer than the control film, with the sucralose microsphere film rated in between and not significantly different from either other treatment. Average hedonic score was higher for the control treatment compared to the quinine and empty microsphere film, while the quinine + sucralose microsphere film did not differ in rating.

## Data Availability

The data used to support the findings of this study are available from the corresponding author upon request.

## Supplementary Materials

Supplementary Materials Supplemental Figure 1: the overall perceived chemosensory intensity and taste quality responses of edible taste strips with no microspheres as a function of time after the strips dissolved (formulation 1). These results indicated that mean taste intensity was in the moderate range and was nearly constant for the first thirty seconds after the strips dissolved. In addition, sweet taste was the predominant taste quality during this time. Finally, the mean hedonic value for strip formulation one was between weakly like and moderately like. These findings indicate that sucralose was rapidly released from strips into the oral cavity. Taken together, these psychophysical results suggest that the edible film formulation used in this study should be useful for masking bitter taste, and is a useful platform for embedding lipid microspheres that encapsulate bitter tasting compounds.
