# Supplementary material for: Toward Improving Medication Adherence: The Suppression of Bitter Taste in Edible Taste Films
Source: Adv Pharmacol Sci. 2018 Jun 25;2018:8043837. doi: 10.1155/2018/8043837 (PMC6036852; doi:10.1155/2018/8043837)
Supplement: Supplementary Materials — Supplemental Figure 1: the overall perceived chemosensory intensity and taste quality responses of edible taste strips with no microspheres as a function of time after the strips dissolved (formulation 1). These results indicated that mean taste intensity was in the moderate range and was nearly constant for the first thirty seconds after the strips dissolved. In addition, sweet taste was the predominant taste quality during this time. Finally, the mean hedonic value for strip formulation one was between weakly like and moderately like. These findings indicate that sucralose was rapidly released from strips into the oral cavity. Taken together, these psychophysical results suggest that the edible film formulation used in this study should be useful for masking bitter taste, and is a useful platform for embedding lipid microspheres that encapsulate bitter tasting compounds. [file 8043837.f1.docx]

**
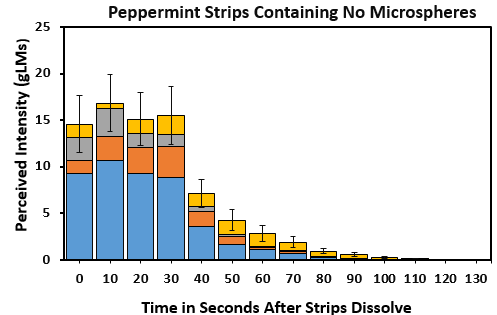
**

**Supplementary Material.**

**A.**

**
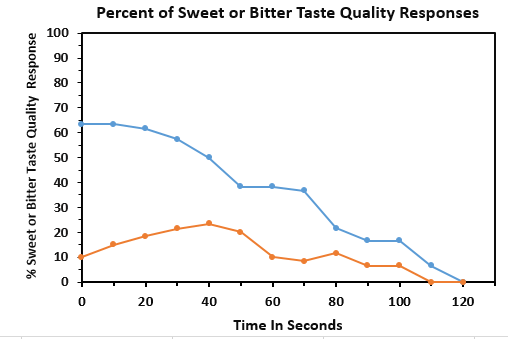
**

**B.**

**Supplemental Figure 1**. Chemosensory properties of edible taste strips with no lipid microspheres. Taste strips contained sucralose and peppermint oil as masking agents in the film base. **A.** Columns represent mean perceived taste intensity as a function of time in seconds after strips dissolve (n = 15). Blue color represents mean sweet taste responses, orange represents bitter taste responses, gray represents other taste, and yellow represents no discernable taste quality. Vertical bars represent standard errors. **B.** Sweet or bitter taste quality response as a function of time in seconds. Blue color represents sweet taste responses, and orange represents bitter taste responses. Mean hedonic score for this strip formulation was +10.3 ±4.4.
